# The Coumarin-Derivative Esculetin Protects against Lipotoxicity in Primary Rat Hepatocytes via Attenuating JNK-Mediated Oxidative Stress and Attenuates Free Fatty Acid-Induced Lipid Accumulation

**DOI:** 10.3390/antiox12111922

**Published:** 2023-10-27

**Authors:** Mengmeng Xia, Zongmei Wu, Junyu Wang, Manon Buist-Homan, Han Moshage

**Affiliations:** 1 Department of Gastroenterology and Hepatology, University Medical Center Groningen, University of Groningen, 9713 GZ Groningen, The Netherlands; m.m.xia@umcg.nl (M.X.); wuzongmei25@outlook.com (Z.W.); j.wang03@umcg.nl (J.W.); m.buist-homan@umcg.nl (M.B.-H.); 2 Department of Laboratory Medicine, University Medical Center Groningen, University of Groningen, 9713 GZ Groningen, The Netherlands

**Keywords:** esculetin, metabolic dysfunction associated fatty liver disease, fatty acids, lipotoxicity, lipid accumulation, lipid droplet, reactive oxygen species, oxidative stress, AMP-activated protein kinase, C-Jun N terminal kinase

## Abstract

Coumarin derivates have been proposed as a potential treatment for metabolic-dysfunction-associated fatty liver disease (MAFLD). However, the mechanisms underlying their beneficial effects remain unclear. In the present study, we explored the potential of the coumarin derivate esculetin in MAFLD, focusing on hepatocyte lipotoxicity and lipid accumulation. Primary cultures of rat hepatocytes were exposed to palmitic acid (PA) and palmitic acid plus oleic acid (OA/PA) as models of lipotoxicity and lipid accumulation, respectively. Esculetin significantly reduced oxidative stress in PA-treated hepatocytes, as shown by decreased total reactive oxygen species (ROS) and mitochondrial superoxide production and elevated expression of antioxidant genes, including Nrf2 and Gpx1. In addition, esculetin protects against PA-induced necrosis. Esculetin also improved lipid metabolism in primary hepatocytes exposed to nonlipotoxic OA/PA by decreasing the expression of the lipogenesis-related gene Srebp1c and increasing the expression of the fatty acid β-oxidation-related gene Ppar-α. Moreover, esculetin attenuated lipid accumulation in OA/PA-treated hepatocytes. The protective effects of esculetin against lipotoxicity and lipid accumulation were shown to be dependent on the inhibition of JNK and the activation of AMPK, respectively. We conclude that esculetin is a promising compound to target lipotoxicity and lipid accumulation in the treatment of MAFLD.

## 1. Introduction

Metabolic-dysfunction-associated fatty liver disease (MAFLD) is currently the most common liver disease. It is characterized by the presence of fat accumulation in the liver in the absence of (excessive) alcohol consumption [1,2]. MAFLD is associated with disturbed lipid metabolism and high levels of (circulating) free fatty acids (FFAs). This results in lipotoxicity. Lipotoxicity is defined as the deleterious effects of exposure to excessive levels of toxic lipid species such as saturated fatty acids (SFAs) [3] and is considered an important cause of the onset and progression of MAFLD [4]. For these reasons, therapeutic strategies for the treatment of MAFLD that target lipotoxicity are under investigation.

Lipotoxicity can result in oxidative stress, the imbalance between the generation of reactive oxygen species (ROS) and antioxidant systems that scavenge ROS [5,6]. ROS are harmful to cells and are generated during the metabolism of drugs like paracetamol and diclofenac and in mitochondria, e.g., in β-oxidation of free fatty acids, relevant in the context of MAFLD, and in the electron transport chain. Moreover, the metabolism of processed food and/or alcohol as well as environmental factors, typical in a Western lifestyle, can also lead to increased generation of ROS [7]. Biomarkers of oxidative stress are increased in patients with MAFLD [6], and SFAs like PA increase the generation of ROS in hepatocytes [8]. Therefore, lipotoxicity-induced oxidative stress has emerged as a therapeutic target for metabolic disorders [4,9]. Excess ROS also promotes inflammation by inducing the synthesis and secretion of various proinflammatory cytokines by both (damaged) hepatocytes and inflammatory cells in the liver, e.g., Kupffer cells [10,11]. This inflammatory response can promote the progression of metabolic diseases like MAFLD [12]. ROS interacts with various transcription factors and/or signaling pathways, such as C-Jun N-terminal kinase, a serine/threonine kinase belonging to the mitogen-activated protein kinase (MAPK) family [13]. The crosstalk between JNK and ROS aggravates the inflammatory response and contributes to organellar dysfunction, especially mitochondrial dysfunction and ER stress, ultimately leading to cell death [13,14,15]. Nuclear factor erythroid-derived 2-like 2 (Nrf2) is a key regulator of the antioxidant response, both under physiological and pathological conditions [16,17], and pharmacological activation of the antioxidant transcription factor Nrf2 prevents steatohepatitis in experimental models of MAFLD via the reduction in oxidative stress [18].

Hepatocytes accumulate lipids as droplets in MAFLD [19]. The lipid droplets (LDs) sequester the excessive supply of FFAs as triglycerides (TGs) [20,21]. In addition, FFAs can be metabolized by β-oxidation in mitochondria [22]. LDs are highly dynamic organelles that can vary in number and size and that store TGs and other lipids [20,21]. However, excessive lipid droplet formation is detrimental [23]. SFAs like PA induce the formation of few and small LDs in hepatocytes and mitochondrial dysfunction, whereas unsaturated FAs (USFAs) like oleate induce the formation of abundant and large LDs and do not compromise mitochondrial function [24]. Therefore, it appears that LD formation, to some extent, protects against lipotoxicity by sequestering FFAs. AMP-activated protein kinase (AMPK) is a key regulator of lipid metabolism because it regulates the activity of enzymes and transcription factors involved in lipid metabolism, such as acetyl-CoA carboxylases (Accs) and sterol regulatory element-binding transcription factor 1/2 (Srebp1/2) [25,26]. Activated AMPK attenuates lipid accumulation by promoting fatty acid β-oxidation and lipophagy [26].

Bioactive compounds derived from medicinal plants have been considered for the treatment of a wide variety of (chronic) diseases for a very long time [27,28,29]. Esculetin is a coumarin-like compound extracted from the plant *Cortex Fraxini* [30,31]. Esculetin has been reported to have protective effects against tumorigenesis, inflammation, and oxidative stress [32]. Furthermore, esculetin has beneficial effects on metabolic dysfunction: it attenuates insulin resistance and vascular dysfunction in diabetic rat models [33,34]. Esculetin also ameliorates hepatic inflammation and lipid metabolism in diet-induced animal models of metabolic dysfunction [35,36]. Esculetin, like other coumarin-like compounds, is rapidly absorbed from the intestine after oral intake and reaches the liver via the portal vein [37,38,39,40]. It is most likely taken up by hepatocytes via passive diffusion [41], although detailed information about its cellular uptake remains scarce. Previously, we have shown that esculetin attenuates proliferation and matrix production of activated hepatic stellate cells, the main cell type involved in liver fibrosis [42]. Together, these effects of esculetin suggest beneficial effects of esculetin in the treatment of MAFLD. However, the protective effects of esculetin, in particular against lipotoxicity, have not been elucidated yet.

The present study was designed to investigate the protective potential of the coumarin-like compound esculetin against lipotoxicity, and in the affirmative, to elucidate the mechanisms underlying these protective effects.

## 2. Materials and Methods

### 2.1. The Animals, Primary Hepatocyte Isolation, and Cell Culture

Male Wistar rats (150–250 g) were purchased from Charles River Laboratories Inc. (Wilmington, MA, USA) and housed in the Central Animal Facility of the University Medical Center Groningen. All animals had ad libitum access to food and water and all animal experiments were approved by the Dutch Central Committee for Animal Experiments and the Animal Welfare Body of the University of Groningen (No. 2115139-01-001). Primary hepatocytes were isolated from rat livers using two-step collagenase perfusion methods as previously described [43] and cultured in William’s E medium (Thermo Fisher Scientific, Breda, The Netherlands) supplemented with 5% fetal bovine serum (Thermo Fisher Scientific), 50 µg/mL gentamicin (Thermo Fisher Scientific), 100 units/mL penicillin (Thermo Fisher Scientific), 10 µg/mL streptomycin (Thermo Fisher Scientific), and 250 ng/mL fungizone (Thermo Fisher Scientific) at 37 °C in a humidified atmosphere containing 5% CO_2_. Only hepatocyte isolations with a viability of more than 80% on Trypan Blue exclusion assay were used.

### 2.2. Chemicals

Esculetin (purity >98%) was purchased from Johnson Matthey company (London, UK). JNK inhibitor II SP600125, AMPKα inhibitor Compound C, sodium palmitate, sodium oleate, bovine serum albumin (fatty-acid free grade), and reduced glutathione ethyl ester were purchased from Sigma-Aldrich (Zwijndrecht, The Netherlands).

### 2.3. Fatty Acid Preparation

Sodium palmitate (Sigma-Aldrich) and oleate (Sigma-Aldrich) were conjugated in 10% bovine serum albumin solution (Sigma-Aldrich, fatty acid free). Then, 20 mmol/L stock solutions of palmitate and oleate were dissolved in William’s E medium without serum to final concentrations of 1 mmol/L (PA) and 750 µmol/L (OA/PA, oleate and palmitate, 2:1).

### 2.4. Detection of Intracellular Lipid Content and Cellular Triglycerides

Oil Red O and Bodipy-LD staining were used to visualize lipids in hepatocytes. Hepatocytes were cultured on coverslips in 12-well plates and exposed to PA and OA/PA for 24 h. After treatment, hepatocytes were first fixed with 3.7% paraformaldehyde solution (Merck Darmstadt, Germany) for 15 min and washed 3 times with PBS (Thermo Fisher Scientific). For Oil Red O staining, hepatocytes were washed 2 times with 60% isopropanol, stained with Oil Red O solution for 10–15 min, and imaged by a slide scanner (Hamamatsu Photonics, Shizouka, Japan). For Bodipy-LD staining, Bodipy dye (1:2500 dilution, Thermo Fisher Scientific) was used. Cells were stained for 15 min and washed 3 times with PBS, then counterstained with 4′,6-diamidino-2-phenylindole (DAPI, Sigma-Aldrich) for 10 min, washed 3 times with PBS (Thermo Fisher Scientific), and visualized using a fluorescent microscope (Leica, Amsterdam, The Netherlands). Cellular triglycerides were measured using a Triglyceride Colorimetric assay kit (Abcam, Cambridge, UK) according to the manufacturer’s instructions.

### 2.5. Viability and Cell Death Assays

Necrotic hepatocytes were evaluated using SYTOX green probe (Thermo Fisher Scientific). Sytox green can only penetrate cells with disrupted plasma membranes, characteristic of necrotic cells. After incubation for 15 min, fluorescent nuclei were observed using a microscope (Leica) at 450–490 nm.

The viability of hepatocytes was also measured using Cell proliferation Reagent WST-1 (Roche, Almere, The Netherlands). Hepatocytes were seeded in 12-well plates and exposed to different concentrations of esculetin for 24 h. After treatment, 10 µL of WST-1 solution per 100 µL medium was added to each well and incubation was continued for 2 h at 37 °C. The formazan dye formed is representative for cell viability and was quantified using a microreader (Bio-Tek, Winooski, VT, USA).

The release of lactate dehydrogenase (LDH) from hepatocytes, an indicator of necrosis, was detected as described previously [44]. In brief, supernatant and cell lysate were collected after treatment and then incubated with LDH reaction buffer. The absorbance was measured at 450 nm using a microreader (Bio-Tek). Results are shown as percentage of LDH released in medium compared to total LDH (in medium and cell lysate).

### 2.6. Measurement of Intracellular Reactive Oxygen Species (ROS) Generation

The production of mitochondrial superoxide was detected using a MitoSOX fluorescent probe (Thermo Fisher Scientific). Briefly, hepatocytes were seeded in 96-well plates and incubated with 2.5 µmol/L MitoSOX for 30 min at 37 °C after treatment. The fluorescence was detected by a microreader (Bio-Tek) with an emission wavelength of 580 nm and an excitation of 480 nm. For immunofluorescent staining of mitochondria-specific ROS, hepatocytes were seeded in 12-well plates with coverslips, incubated with 2.5 µmol/L MitoSOX for 15 min at 37 °C, followed by staining with DAPI for 10 min. Subsequently, hepatocytes were washed 3 times with PBS. Images were acquired using a fluorescent microscope (Leica).

Cellular ROS was assessed by 2,7-dichlorofluorescein diacetate (DCFH/DA, Abcam) assay. Hepatocytes were seeded in 96-well plates and stained with DCFH/DA for 45 min at 37 °C in the dark and fluorescence was visualized by a fluorescent microscope (Leica).

### 2.7. Real-Time Quantitative Polymerase Chain Reaction (RT-qPCR)

Total RNA was extracted from hepatocytes using TRI reagent (Sigma-Aldrich) following the manufacturer’s protocols. A NanoDrop spectrophotometer (Thermo Fisher Scientific) was used to determine the quality and quantity of RNA. cDNA synthesis was prepared using M-MLV reverse transcriptase (Thermo Fisher Scientific). qPCR was performed using a QuantStudioTM 3 system (Thermo Fisher Scientific) with Taqman primers and probes listed in Table 1. The qPCR reaction was a first step at 95 °C for 10 min, followed by 40 cycles of 95 °C for 15 s and 95 °C for 1 min. The relative expression levels were calculated by using the 2^−ΔΔCt^ method and 18S was used for normalizing mRNA level. All samples were analyzed in duplicate.

### 2.8. Western Blot Analysis

Total protein was harvested from hepatocytes after treatment and quantified using a Bio-Rad protein assay kit (Bio-Rad, Veenendaal, The Netherlands). Equivalent amounts of protein were separated by 10% SDS-PAGE gels and transferred to nitrocellulose membranes (Amersham, Piscataway, NJ, USA) using a Trans-Blot Turbo Blotting system (Bio-Rad). Membranes were incubated with primary antibodies as follows: anti-phospho-AMPKα (#2531, Cell Signaling Technology, Leiden, The Netherlands, 1:1000), anti-AMPKα (#2532, Cell Signaling Technology, 1:1000), anti-phospho-SAPK/JNK (#9251, Cell Signaling Technology, 1:1000), anti-SAPK/JNK (#9252, Cell Signaling Technology, 1:1000), anti-Nrf2 (ab31163, Abcam, 1:1000), anti-GAPDH (CB1001, Calbiochem, Amsterdam, The Netherlands, 1:10,000), and anti-β-actin (#4970, Cell Signaling Technology, 1:1000). Horseradish peroxidase-conjugated polyclonal goat-anti rabbit (P0448, Dako, Glostrup, Denmark, 1:1000) and rabbit-anti-mouse (P0260, Dako, 1:1000) were used as secondary antibodies. Protein signals were detected using a ChemiDoc MP Imaging system (Bio-Rad).

### 2.9. Statistical Analysis

All data are presented as means ± SD. All experiments were repeated at least three times using hepatocytes from different isolations. Statistical analysis was performed using GraphPad Prism (V.8.0.1, GraphPad Software, San Diego, CA, USA). One-way ANOVA test followed by Tukey’s multiple comparison tests were used to evaluate differences between groups. *p* values < 0.05 were considered statistically significant.

## 3. Results

### 3.1. Esculetin Attenuates Palmitic-Acid-Induced Toxicity and Necrosis in Primary Rat Hepatocytes

**Figure 1:**
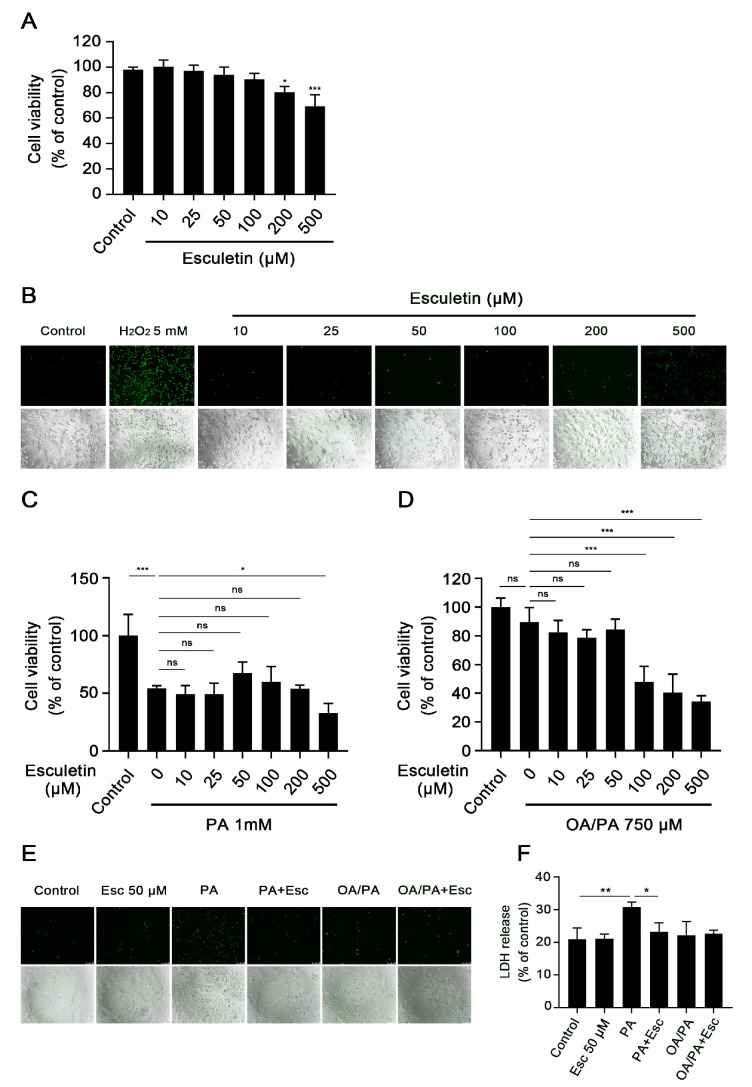
Esculetin attenuates PA-induced toxicity and necrosis in primary rat hepatocytes. (**A**,**B**) Primary rat hepatocytes were incubated with the indicated doses of esculetin for 24 h and cell viability was determined using WST-1 assay (**A**) and Sytox Green nuclear staining (**B**). Hydrogen peroxide (H_2_O_2_) was used as a positive control for necrosis. Magnification 10×. (**C**,**D**) Primary rat hepatocytes were treated with PA or OA/PA alone and in combination with different doses of esculetin for 24 h. Cell viability was measured by WST-1 assay. (**E**,**F**) Primary rat hepatocytes were treated with PA or OA/PA alone or with esculetin (50 µmol/L) for 24 h. Cell necrosis was detected by SYTOX Green staining (**E**) and quantified by LDH assay (**F**), respectively. Magnification 10×. Data are expressed as mean ± SD (*n* ≥ 3 for each group), * *p* < 0.05 (significant difference), ** *p* < 0.01, *** *p* < 0.001, ns (no significant difference between indicated groups).

We first evaluated the cytotoxicity of esculetin on primary rat hepatocytes. Primary rat hepatocytes were incubated with different concentrations of esculetin (from 10 µmol/L to 500 µmol/L) for 24 h, followed by SYTOX green nuclear staining and WST-1 assay to determine cell toxicity. WST-1 assay revealed that esculetin did not affect hepatocyte viability at a range of 10 µmol/L to 100 µmol/L, whereas a significant reduction in viability was observed when the concentration of esculetin was more than 100 µmol/L (Figure 1A). Consistent with this result, high doses (over 100 µmol/L) but not low doses (10 µmol/L to 100 µmol/L) of esculetin induced necrotic cell death in hepatocytes, as shown by the increased number of SYTOX green positive cells (Figure 1B). Considering the toxic effects of high concentrations of esculetin on PA and OA/PA-induced hepatocytes (Figure 1C and 1D, respectively), 50 µmol/L of esculetin was used in all following experiments.

PA alone caused significant necrosis in hepatocytes, as reflected by increased nuclear SYTOX green staining and high LDH leakage (Figure 1E and 1F, respectively), consistent with our previous reports [45,46]. Esculetin treatment significantly attenuated necrosis in PA-treated hepatocytes (Figure 1E,F). In contrast, OA/PA alone or together with esculetin did not induce significant hepatocyte necrosis (Figure 1E,F).

### 3.2. Esculetin Attenuates Palmitic Acid-Induced Oxidative Stress in Primary Rat Hepatocytes

**Figure 2:**
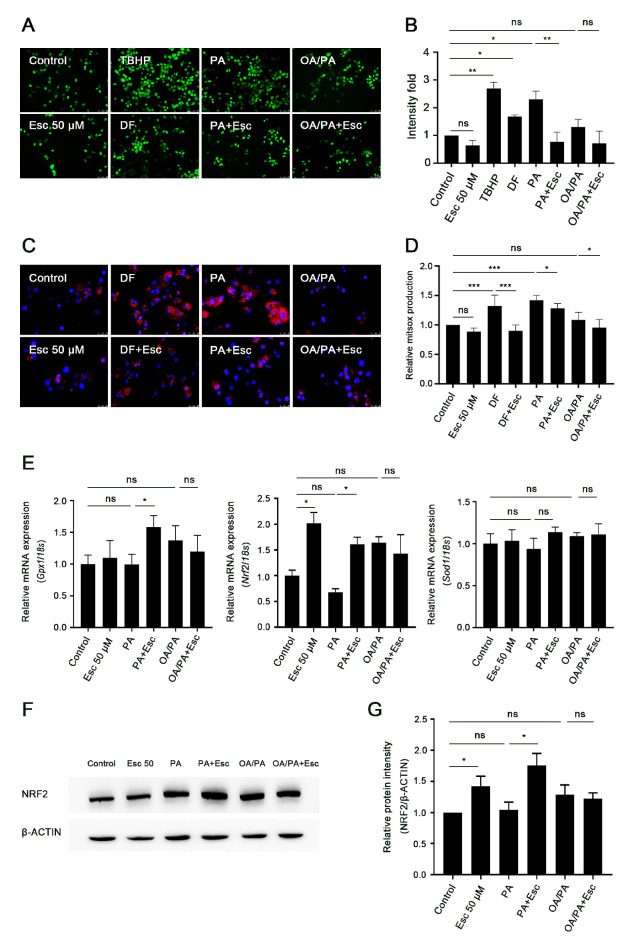
Esculetin attenuates PA-induced oxidative stress in primary rat hepatocytes. Primary rat hepatocytes were pretreated with esculetin (50 µmol/L) for 30 min and then exposed to PA or OA/PA for 4–6 h. (**A**,**B**) Total cellular ROS generation in primary hepatocytes was measured by DCFH/DA assay. Tert-Butyl hydroperoxide (TBHP) and diclofenac (DF) served as the positive controls for ROS generation. Magnification 10×. (**C**,**D**) Mitochondrial superoxide anion generation in primary hepatocytes was detected using MitoSOX assay. (**C**) MitoSOX staining in red, nuclei stained with DAPI in blue. Magnification 40×. (**D**) Values of MitSOX were measured using a microreader. DF was used as positive control. (**E**) Relative mRNA expression of oxidative-stress-related genes including Gpx1, Nrf2, and Sod1 were measured using RT-qPCR and normalized to 18s level. (**F**,**G**) Protein expression of NRF2 was evaluated using Western blot analysis and analyzed using ImageJ 1.53e. All data are expressed as mean ± SD (*n* ≥ 3 in each group), ns, no significant difference, * *p* < 0.05, ** *p* < 0.01, *** *p* < 0.001.

Exposure to saturated fatty acids (SFAs) like palmitic acid (PA) can induce the generation of ROS in primary hepatocytes [8]. Since esculetin has been reported to have antioxidant effects [31,47], we evaluated the effects of esculetin on PA-induced ROS production. DCFH/DA staining revealed that exposure to PA, but not OA/PA, induced significant ROS generation in hepatocytes (Figure 2A,B). Esculetin significantly decreased PA-induced ROS generation (Figure 2A,B). Since mitochondria are the major source of ROS production [48], we next investigated whether esculetin affects mitochondrial ROS generation. MitoSOX assay demonstrated that esculetin effectively inhibited mitochondrial superoxide anion generation induced by PA treatment (Figure 2C,D). Similar to the results obtained with the DCFH/DA assay, OA/PA alone or in combination with esculetin did not result in excessive mitochondrial superoxide formation in hepatocytes compared to the control group (Figure 2C,D). Consistent with these findings, esculetin significantly increased the expression of antioxidant genes (Nrf2 and Gpx1, not Sod1) in the presence of PA, but not in the presence of OA/PA (Figure 2E–G).

### 3.3. Esculetin Attenuates JNK Activation in Palmitic-Acid-Treated Hepatocytes

**Figure 3:**
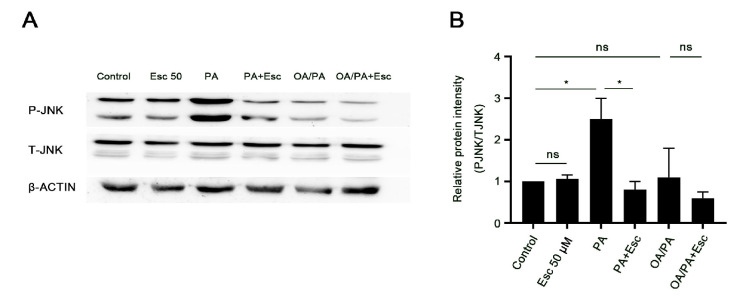
Esculetin attenuates JNK activation in PA-treated primary rat hepatocytes. (**A**) Primary rat hepatocytes were exposed to PA or OA/PA with or without esculetin (50 µmol/L). After 6 h, total and phosphorylated JNK levels were assayed by Western blot analysis. (**B**) Relative JNK protein expression was analyzed using ImageJ 1.53e. All data are expressed as mean ± SD (*n* ≥ 3 in each group), ns, no significant difference, * *p* < 0.05.

C-Jun N terminal kinase (JNK) is an important signaling molecule that mediates cell inflammation and cell death in liver injury [49,50]. We evaluated JNK activation in PA- and OA/PA-treated hepatocytes using Western blot analysis for phosphorylated (activated) JNK. PA treatment, but not OA/PA treatment, significantly increased the phosphorylation of JNK (Thr183/Tyr185) in hepatocytes (Figure 3A,B). Esculetin treatment significantly reduced PA-stimulated phosphorylation of JNK.

### 3.4. JNK Activation Induced by Palmitic Acid Leads to Increased Oxidative Stress and Lipotoxicity in Hepatocytes

Both JNK activation and oxidative stress are regarded as important mechanisms for lipotoxicity [51,52]. However, there is a complex relationship or cross-talk between JNK activation and oxidative stress. To elucidate the inter-relationship between JNK activation and oxidative stress in esculetin-mediated protection against lipotoxicity, we used SP600125 (a JNK-specific inhibitor) to block JNK activation and the antioxidant glutathione mono-ethyl ester (GSH-MEE), a cell-permeable glutathione precursor.

**Figure 4:**
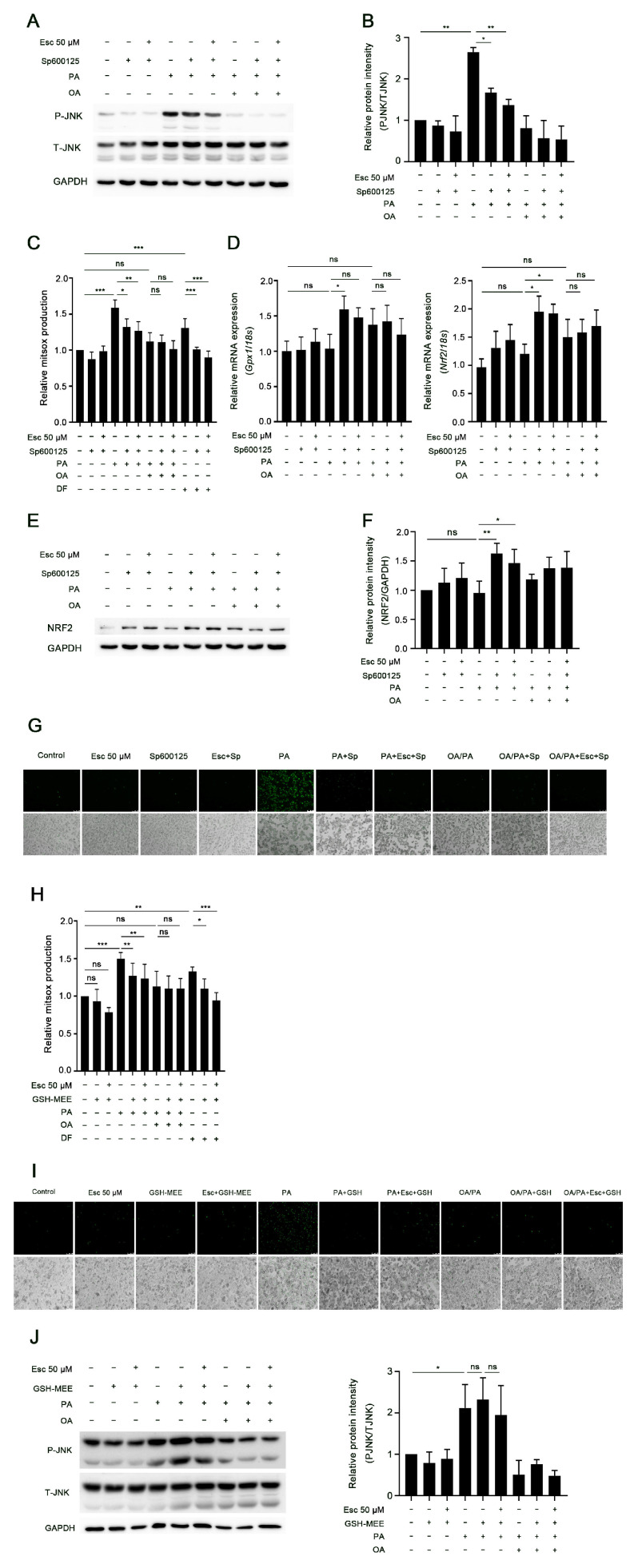
JNK activation induced by palmitic acid leads to increased oxidative stress and lipotoxicity in hepatocytes. Hepatocytes were treated with PA or OA/PA for 6 h with or without the JNK inhibitor SP600125 and/or esculetin. (**A**,**B**) Total and phosphorylated protein levels of JNK after treatment with PA, OA/PA, SP600125, and esculetin were detected by Western blot analysis and quantified by ImageJ 1.53e. GAPDH served as the loading control. (**C**) Mitochondrial superoxide production was measured using MitSOX assay. (**D**) Relative mRNA levels of antioxidant genes (Gpx1 and Nrf2) were assayed using RT-qPCR and normalized to 18s. (**E**,**F**) Western blot analysis of NRF2 protein level in PA- or OA/PA-treated hepatocytes with or without SP600125 and/or esculetin and relative NRF2 expression was analyzed by ImageJ 1.53e. (**G**) Necrotic hepatocytes were determined using SYTOX Green assay. Magnification 10×. (**H**–**J**) Primary rat hepatocytes were treated with the antioxidant GSH-MEE (cell-permeable glutathione precursor) and PA or OA/PA for 6 h with or without esculetin. (**H**) Mitochondrial superoxide production in PA- or OA/PA-treated hepatocytes with or without GSH-MEE determined using MitSOX assay. (**I**) Hepatocyte necrosis detected using SYTOX Green nuclear staining. Magnification 10×. (**J**) Total and phosphorylated protein levels of JNK in PA- or OA/PA-treated hepatocytes with or without GSH-MEE and quantified by ImageJ 1.53e. The data are presented as mean ± SD (*n* ≥ 3 in each group), ns, not significant, * *p* < 0.05, ** *p* < 0.01, *** *p* < 0.001.

As illustrated in Figure 4A,B, SP600125, as expected, attenuated the phosphorylation of JNK (Thr183/Tyr185) in response to PA treatment. This inhibition was even more pronounced in the presence of esculetin. In line with these results, SP600125 also decreased PA-induced mitochondrial superoxide generation (Figure 4C), suggesting that SP600125 suppressed PA-induced oxidative stress in hepatocytes. In addition, SP600125 increased mRNA and protein expression of the antioxidant response gene Nrf2 and mRNA expression of Gpx1 only in PA-treated hepatocytes (Figure 4D–F). SP600125 also reduced PA-induced hepatocyte necrosis (Figure 4G). The phosphorylation of JNK (Thr183/Tyr185), the generation of mitochondrial superoxide anions, the expression levels of Nrf2 and Gpx1, and cell necrosis were not affected by OA/PA alone or in combination with SP600125 or esculetin (Figure 4A–G).

The antioxidant GSH-MEE reduced PA-induced mitochondrial superoxide anion generation and necrosis (Figure 4H,I), but it did not reverse the PA-induced phosphorylation of JNK (Thr183/Tyr185) (Figure 4J). Again, mitochondrial superoxide anion generation, cell necrosis, and the phosphorylation of JNK (Thr183/Tyr185) were not affected by GSM-MEE in OA/PA-treated hepatocytes (Figure 4H–J). Collectively, these results suggest that PA-induced lipotoxicity depends on JNK activation and that the increased oxidative stress is a downstream effect of JNK activation.

### 3.5. Esculetin Improves Free Fatty Acid-Induced Lipid Accumulation and Metabolism

**Figure 5:**
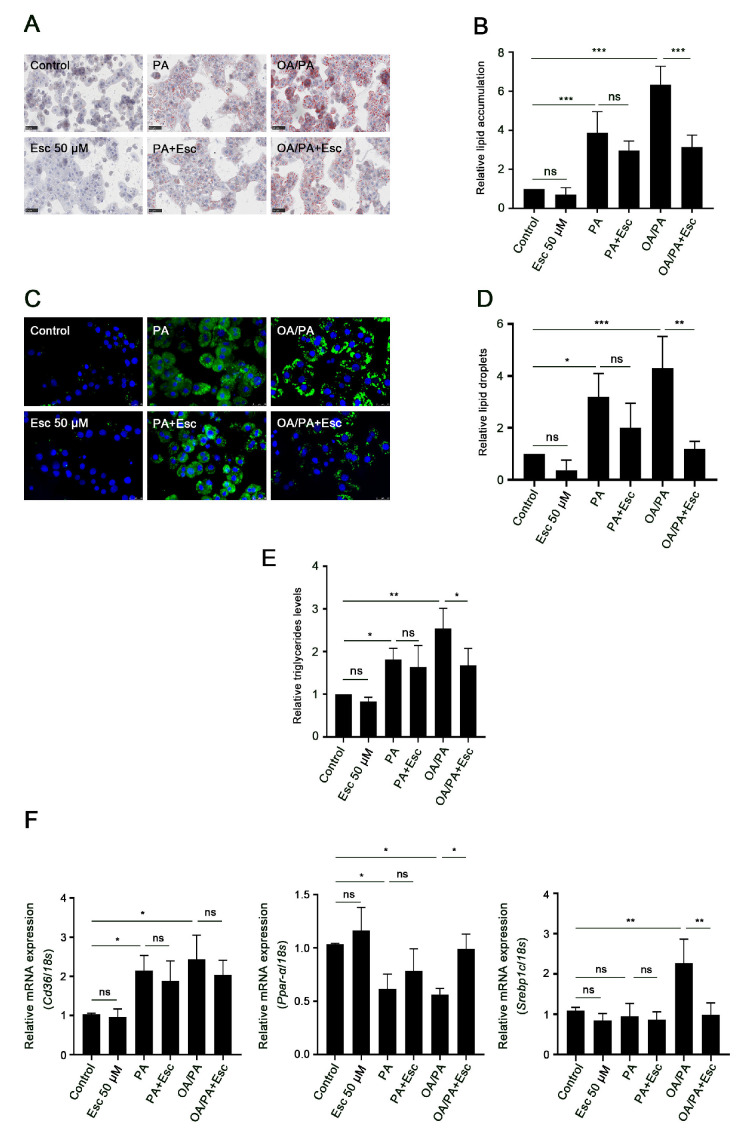
Esculetin attenuates free fatty acid-induced lipid accumulation and metabolism. Primary rat hepatocytes were treated with PA or OA/PA for 24 h in the presence or absence of esculetin. (**A**,**B**) Oil Red O staining was used to visualize lipid accumulation, and the degree of lipid accumulation was evaluated by using Image Pro Plus 6.0. Magnification 20×, scale bars 50 μm. (**C**,**D**) LDs in hepatocytes were visualized with BODIPY-LD dye (green) and nuclei were stained with DAPI (blue). The fluorescence was measured using Image Pro Plus 6.0. Magnification 40×. (**E**) Triglyceride contents in hepatocytes with indicated treatments. (**F**) Relative mRNA levels of lipid metabolism-related genes, including the lipid uptake gene Cd36, the FA β-oxidation-related gene Ppar-α, and the lipogenesis-related gene Srebp1c in primary hepatocytes. Data are mean ± SD (*n* ≥ 3 in each group), ns, no significant difference, * *p* < 0.05, ** *p* < 0.01, *** *p* < 0.001.

Next, we evaluated the impact of esculetin on PA and OA/PA-induced lipid accumulation in hepatocytes. Oil Red O and Bodipy-LD staining showed that although both PA and OA/PA induced lipid droplets (LDs) accumulation in hepatocytes, OA/PA treatment induced significantly more and larger-sized LDs than PA treatment (Figure 5A–D). Consistent with these results, TG content in OA/PA-treated hepatocytes was significantly increased compared with PA-treated hepatocytes (Figure 5E). Additionally, esculetin treatment markedly decreased LDs accumulation and TG content in OA/PA-treated hepatocytes, but not in PA-treated hepatocytes (Figure 5A–E). We next determined the expression of genes involved in lipogenesis (Srebp1c), FA β-oxidation (Ppar-α), and lipid transport (Cd36). As shown in Figure 5F, the expression of Cd36 was increased in both PA- and OA/PA-treated groups, whereas the expression of Ppar-α was decreased by both PA and OA/PA treatment. In addition, significantly increased expression of Srebp1c was observed in OA/PA-treated hepatocytes but not in PA-treated hepatocytes, compared to nontreated control hepatocytes. Esculetin decreased the expression of Srebp1c and increased the expression of Ppar-α, but had no effect on the expression of Cd36 in OA/PA-treated hepatocytes (Figure 5F). Esculetin had no effect on the expression of Srebp1c, Ppar-α, and Cd36 in PA-treated hepatocytes (Figure 5F). These data suggest that esculetin attenuates OA/PA-induced lipid accumulation.

### 3.6. Esculetin Promotes AMPKα Phosphorylation in Free Fatty Acid-Treated Hepatocytes

**Figure 6:**
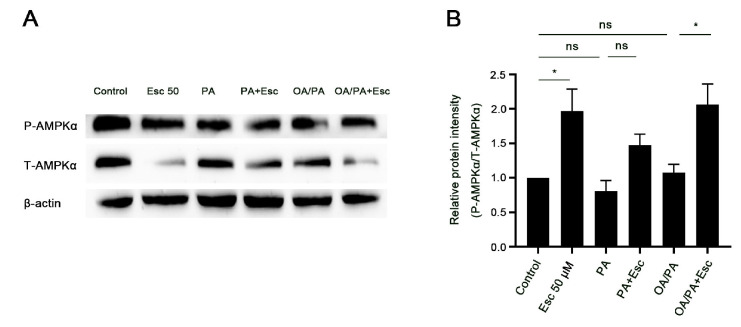
Esculetin promotes AMPKα phosphorylation in free fatty acid treated hepatocytes. (**A**,**B**) Primary rat hepatocytes were exposed to PA or OA/PA for 24 h with or without esculetin treatment. AMPKα activation was determined using Western blot analysis (**A**) and the relative protein density was analyzed by ImageJ 1.53e (**B**). β-ACTIN was the loading control. Data are mean ± SD (*n* ≥ 3 in each group), ns, no significant difference, * *p* < 0.05.

It has been reported that esculetin restored activation of AMPKα in H_2_O_2_-treated human aortic endothelial cells and in OA/PA-treated human hepatoma HepG2 cells [53,54]. AMPKα is a key sensor in lipid homeostasis by modulating the expression of genes involved in lipogenesis and FA oxidation [26]. Therefore, we determined the activation of AMPKα in PA- and OA/PA-treated hepatocytes by Western blot analysis. As shown in Figure 6, esculetin markedly increased phosphorylation of AMPKα (Thr172) in OA/PA-treated hepatocytes, but not in PA-treated hepatocytes (Figure 6A,B).

### 3.7. AMPKα Activation Mediates the Effects of Esculetin on Lipid Accumulation but Not on Oxidative Stress and Necrosis

**Figure 7:**
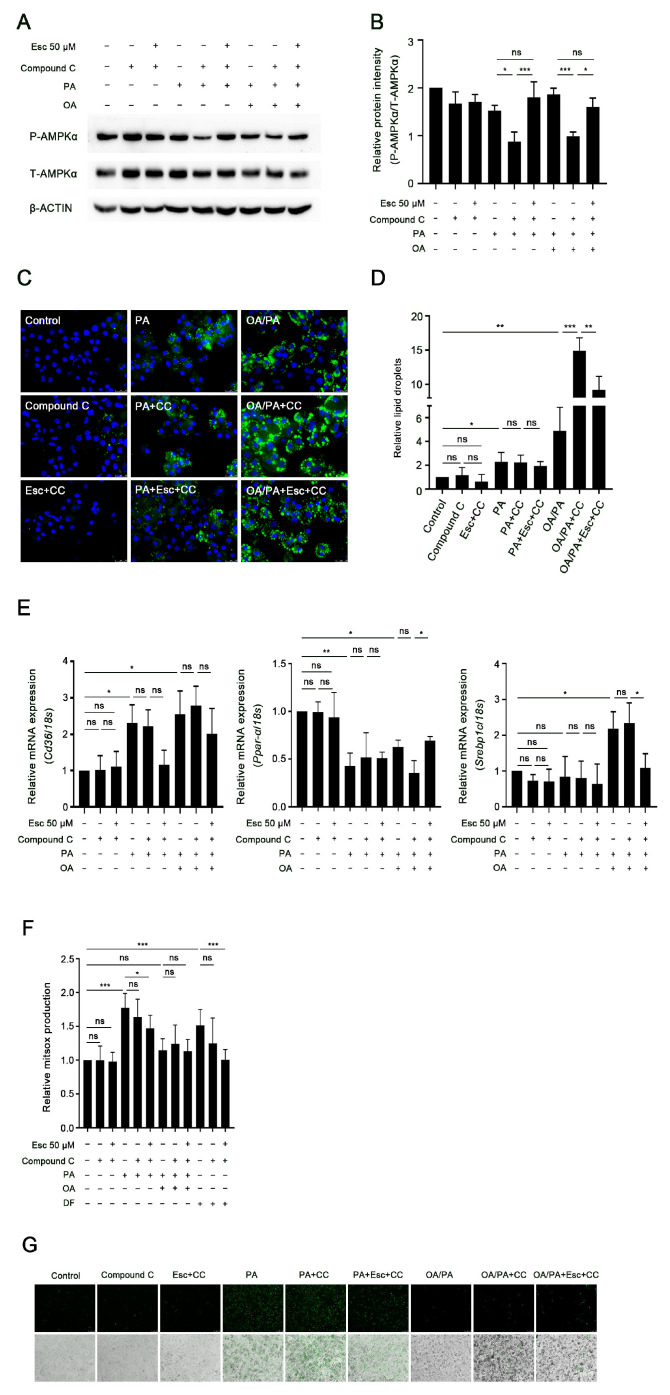
AMPKα activation mediates the effects of esculetin on lipid accumulation but not on oxidative stress and necrosis. (**A**,**B**) AMPKα phosphorylation was detected using Western blot assay and quantified by ImageJ 1.53e. (**C**,**D**) LDs in Compound C-treated hepatocytes in the presence or absence of FFAs and/or esculetin were detected by BODIPY-LD staining. Magnification 40×. Relative fluorescence was evaluated by Image Pro Plus 6.0. (**E**) Relative mRNA levels of genes involved in lipogenesis and FA oxidation genes were detected by RT-qPCR. (**F**) Mitochondrial superoxide production in the indicated groups. (**G**) Necrosis in Compound C-treated hepatocytes with and without OA/PA or PA treatment was evaluated using SYTOX Green assay. Magnification 10×. Data are presented as mean ± SD (*n* ≥ 3 in each group), ns, no significant difference, * *p* < 0.05, ** *p* < 0.01, *** *p* < 0.001.

To further determine whether phosphorylation of AMPKα (Thr172) mediates the effect of esculetin on lipid accumulation and lipotoxicity, the AMPKα specific inhibitor, Compound C (CC), was used. Western blot analysis showed that the phosphorylation of AMPKα in PA- and OA/PA-treated hepatocytes was dramatically decreased by CC, while this effect was abolished by cotreatment with esculetin (Figure 7A,B).

To clarify whether AMPKα is involved in the esculetin-mediated reduction in lipid accumulation in OA/PA-treated hepatocytes, Bodipy-LD staining was performed to examine the lipid contents in the presence of CC. CC caused increased LDs accumulation in OA/PA-treated hepatocytes. Cotreatment with esculetin reversed the increased LDs induced by CC (Figure 7C,D). Treatment with CC alone or with esculetin did not result in significant changes in PA-induced lipid accumulation (Figure 7C,D). To further investigate whether esculetin-mediated lipid metabolism is dependent on AMPKα activation, the expression of lipid-metabolism-related genes was determined. CC decreased the expression of Ppar-α and increased the expression of Srebp1c in OA/PA-induced group. Both changes were reversed by esculetin (Figure 7E).

Next, we investigated whether AMPKα also mediated the protective effects of esculetin against oxidative stress and cell necrosis. CC did not affect the increased ROS production in PA-induced hepatocytes, nor the protective effect of esculetin against PA-induced ROS generation (Figure 7F). Importantly, treatment of CC did not attenuate PA-induced necrosis in hepatocytes (Figure 7G). These data indicate that AMPKα exclusively mediates the effects of esculetin on lipid metabolism.

## 4. Discussion

Emerging evidence suggests that lipotoxicity is the key pathogenic factor in the development of MAFLD and may serve as a potential treatment target [55,56]. The current study uncovered that the coumarin-derivative esculetin protects against lipotoxicity and lipid accumulation induced by fatty acids through two different pathways: (1) esculetin attenuates oxidative stress and cell death in lipotoxic hepatocytes by inhibiting JNK activation, and (2) esculetin reduces lipid overload and improves lipid metabolism in steatotic hepatocytes by upregulating AMPK activation.

Saturated fatty acids (SFAs), especially those with a carbon chain length of 16 or more, such as palmitate (PA, C16:0) and stearate (C18:0), are the main contributors to lipotoxicity. On the other hand, monounsaturated FAs (e.g., oleate, OA) and polyunsaturated fatty acids do not induce cell death [57,58]. OA and PA are the most abundant FAs in both normal subjects and patients with MAFLD [59] and were used in our studies. PA (1 mmol/L) was used to induce lipotoxicity in hepatocytes, whereas the combination OA/PA (750 µmol/L) was used to induce steatosis in hepatocytes.

Lipotoxicity causes oxidative stress because chronic exposure to toxic lipids causes impaired oxidative phosphorylation, a disturbed redox balance, and exacerbation of the production of ROS [6]. ROS overproduction can trigger lipid peroxidation, DNA damage, and protein misfolding, as well as the release of damage-associated molecules and the activation of cell death pathways [60,61]. Total intracellular ROS generation was enhanced in hepatocytes treated with PA, but not in hepatocytes treated with OA/PA. This finding is consistent with previous studies [62], suggesting that SFAs induce ROS accumulation in the liver but unsaturated fatty acids do not. In addition, esculetin attenuated the increased ROS production in PA-exposed hepatocytes. Lipotoxicity is also associated with inflammation [63]. Clinical and experimental studies have revealed that lipotoxic hepatocytes produce increased levels of proinflammatory cytokines and proteins such as iNOS, IL-1β, TNF-α, and IL-6, and that these increased levels correlate with the severity of NASH [64]. Indeed, we observed that PA, but not OA/PA, increased mRNA expression of iNOS and Il-1β in primary hepatocytes and that esculetin decreased the expression of iNOS and Il-1β. Oxidative stress induced by toxic PA results in hepatocyte cell death, mainly via necrosis [45,46]. In contrast, OA/PA treatment of hepatocytes did not induce cell death. We observed that PA-induced necrosis was dependent on oxidative stress, since it could be prevented by treatment with the antioxidant GSH-MEE. Esculetin effectively reversed necrosis induced by PA and also inhibited total ROS production in PA-treated hepatocytes.

Mitochondria are the key regulators of cellular energy metabolism and the principal site of endogenous ROS generation [6,65]. Increased influx of FAs entering mitochondria increases mitochondrial membrane permeability and causes electron leakage from the electron transport chain. This leads to mitochondrial impairment and elevated generation of mitochondrial reactive oxygen species (mtROS). Persistent mtROS production is associated with mitochondrial dysfunction and cell death [66]. In our study, we demonstrate that PA, but not OA/PA, enhanced mtROS generation in primary hepatocytes, consistent with previous reports [67]. Esculetin has been shown to protect mitochondria and reduce mtROS generation in endothelial cells exposed to ROS [54]. Our current study revealed that esculetin effectively reversed mtROS generation in PA-treated hepatocytes and demonstrated the potential role of esculetin in protecting mitochondrial function under conditions of metabolic stress in hepatocytes.

The transcription factor Nrf2 is an important component of the cellular antioxidant system [16,17]. In vivo and in vitro models of MAFLD have shown that pharmacologic activation of Nrf2 reduces ROS production, DNA damage, and programmed cell death, and ameliorates steatosis and fibrogenesis [68,69], indicating that Nrf2 plays a protective role against oxidative-stress-induced injury in MAFLD. Mechanistically, the transcription factor Nrf2 translocates into the nucleus in response to ROS and activates antioxidant genes, including Gpx1, Ho-1, and Sod1/Sod2 [70]. Sod1/Sod2 and Gpx1 are important in the scavenging of mtROS [71]. It has been demonstrated that coumarin-like compounds can bind to Nrf2 with high affinity and promote its activation, thereby contributing to the defense against oxidative stress [72]. In the present study, esculetin increased the expression level of Nrf2 in hepatocytes, both with and without PA stimulation. Esculetin also increased the expression of Gpx1 in hepatocytes treated with PA. These results indicate that esculetin may act as a pharmacological activator of Nrf2.

Sustained JNK activation has been reported in SFA-induced lipotoxicity in MAFLD [73,74]. We observed significant activation of JNK in hepatocytes treated with PA, but not with OA/PA. It is debated if JNK activation leads to increased ROS generation, or if increased ROS generation leads to JNK activation. Using specific inhibitors of JNK and ROS-scavenging antioxidants, we demonstrate that JNK activation causes ROS generation in PA-treated hepatocytes. Esculetin decreased JNK activation and reduced mtROS generation. These results demonstrate that esculetin protects against PA-induced lipotoxicity by inhibiting JNK activation and inducing the Nrf2-mediated antioxidant response, leading to reduced mtROS generation and cell death.

In conditions of FA overload, FAs can be esterified into TG and cholesterol esters and stored as LDs within hepatocytes [75]. LDs are not merely passive storage sites of TGs, but play an active role in cellular homeostasis. Their number, size, and morphology vary in response to different stimuli [24]; differential FAs-induced LDs seem to have different crosstalk with target organelle, e.g., mitochondria [24]. OA promoted the formation of abundant and large LDs proximal to mitochondria. On the other hand, PA promoted the formation of fewer and smaller LDs proximal to mitochondria, resulting in enhanced mitochondrial membrane potential (Δψm) and triggering rapid mitochondrial fragmentation [24]. The sequestration of FAs as TGs in LDs has been proposed as a mechanism to prevent FA toxicity in hepatocytes, and USFAs like oleate are more prone to be esterified and stored in LDs than SFAs like palmitate [56]. In the current study, esculetin treatment decreased LDs accumulation in OA/PA-treated hepatocytes, but not in PA-treated hepatocytes. Since esculetin did not increase toxicity of FFAs, these results suggest that the lipid-lowering effect of esculetin may be due to effects on FA uptake and/or metabolism. Moreover, since esculetin has no effect on LDs formation and morphology in PA-treated hepatocytes, the protective effect of esculetin against lipotoxicity induced by PA is not mediated by LDs.

The transcription factor Srebp1c is a major regulator of lipid metabolism. It promotes lipogenesis by increasing the expression of lipogenic genes such as Fas, Acc1, and Scd-1 [76]. Cd36 is the FA importer and promotes de novo lipogenesis in the liver. Loss of Cd36 in hepatocytes reduces the uptake of FAs and lipid accumulation, thereby improving hepatic steatosis. Conversely, overexpression of Cd36 in the liver leads to the opposite effects, resulting in lipid accumulation [77]. Activation of the transcription factor Ppar-α decreases lipid accumulation and prevents hepatic steatosis [78]. This effect is mediated via the activation of Ppar-α-mediated FA β-oxidation. Ppar-α can also increase lipolysis by inducing its direct target lipoprotein lipase (Lpl) through the PPAR response element (PPRE) [78]. In the present study, esculetin decreased the expression of Srebp1c and increased the expression of Ppar-α in OA/PA-treated hepatocytes, suggesting that the protective role of esculetin against lipid accumulation is most likely due to the suppression of lipogenesis and the activation of FA β-oxidation.

AMPK is a key regulator of energy homeostasis [25,26]. In the liver, AMPK functions as a central metabolic switch that is involved in fatty acid (FA), triglyceride (TG), cholesterol, and glucose metabolism [25,26]. Pharmacologic activation of AMPK has been proposed as a therapeutic target in the context of MAFLD [79]. Phosphorylation of AMPK, in particular the catalytic α subunit, has been shown to decrease lipid droplet formation and to alleviate hepatic steatosis in both diet-induced MAFLD models as well as in hepatocytes treated with FAs [80,81,82]. Previous studies have revealed that coumarin derivatives act as lipid-lowering agents by activating AMPK-regulated lipogenic signaling pathways or AMPK-mediated autophagy [83,84]. Esculetin decreases lipogenesis and lipid uptake in FA-treated hepatocytes by phosphorylating AMPKα at Thr172 and downregulating the expression of the target gene Acc [36,53]. Our study demonstrates that esculetin activates AMPK by phosphorylating AMPKα at Thr172, and this effect of esculetin may be responsible for its lipid-lowering effect. As mentioned, esculetin did not decrease LDs accumulation in PA-treated hepatocytes. This may be due to the fact that the amounts of LDs in PA-treated hepatocytes is already very low in comparison to FA-treated hepatocytes; therefore, the effect of esculetin is not visible.

A limitation of our study is that we performed only in vitro studies, using cultured cells. In order to apply esculetin in clinical practice or as a food supplement, additional in vivo studies are required, focusing on optimizing the mode of administration and dosage, intestinal absorption, organ distribution, cellular uptake mechanisms, and (hepatic) metabolism.

**Figure 8:**
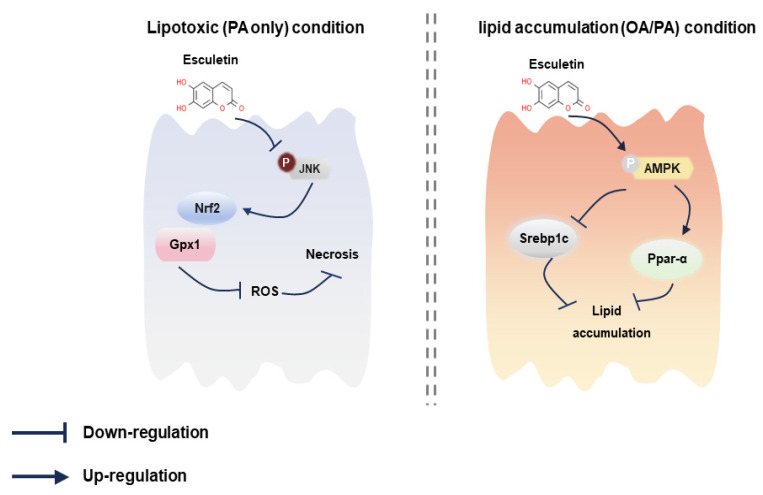
Proposed model of the protective effects of esculetin against lipotoxicity and lipid accumulation. In PA-induced lipotoxicity, esculetin suppressed JNK activation and increased expression of the antioxidant genes Nrf2 and Gpx1, reducing ROS production and cell necrosis. Esculetin also exhibited lipid-lowering effects through AMPKα/Srebp1c/Ppar-α pathway in response to OA/PA treatment.

In conclusion, this study elucidated two different protective mechanisms of esculetin in response to fatty acid exposure (Figure 8). On the one hand, PA induces lipotoxicity, as shown by increased oxidative stress, especially increased mitochondrial superoxide generation, as well as increased hepatocyte necrosis. In this lipotoxic condition, esculetin emerges as the pivotal modulator via inhibiting JNK activation and consequently reducing PA-induced oxidative stress. Esculetin increased the activity of the antioxidant Nrf2-Gpx1 system and inhibited JNK activation, leading to less oxidative stress and ultimately less necrosis. On the other hand, the fatty acid combination OA/PA induces lipid accumulation rather than lipotoxicity. In this condition, esculetin activates AMPKα to improve lipid metabolism, most likely by regulating downstream targets: suppression of Srebp1c expression and increasing Ppar-α expression, thereby inhibiting the formation of lipid droplets. Notably, esculetin did not alter lipid overload in PA-treated hepatocytes, probably because the lipid accumulation induced by PA is very low in comparison to OA/PA. The esculetin-mediated AMPKα activation did not ameliorate lipotoxicity, oxidative stress, and necrosis, suggesting that the protective effect of esculetin against lipotoxicity is independent of its lipid-lowering effect and AMPKα activation. Collectively, esculetin may be a potential therapeutic candidate to treat lipotoxicity in MAFLD.

## Figures and Tables

**Table 1:** Primers and probes sequences.

Gene	Sense 5′–3′	Antisense 5′–3′	Probe 5′–3′	Accession Number
18s	CGGCTACCACATCCAAGGA	CCAATTACAGGGCCTCGAAA	CGCGCAAATT ACCCACTCCCGA	X01117
Srebp1c	GGAGCCATGGATTGCACATT	CCTGTCTCACCCCCAGCATA	CAGCTCATCAACAACCAAGACAGTGACTTCC	XM_213329
Cd36	GATCGGAACTGTGGGCTCAT	GGTTCCTTCTTCAAGGACAACTTC	AGAATGCCTCCAAACACAGCCAGGAC	NM_031561
Pparα	CACCCTCTCTCCAGCTTCCA	GCCTTGTCCCCAC ATATTCG	TCCCCACCAGTACAGATGAGTCCCCTG	NM_013196
Nrf2	AGCCCAGCACATCCAGACA	TGTCTCTGCCAAAAGCTGCAT	TCAGCTACTCCCAGGTTGCCCACATTC	NM_031789
Sod1	CAGGACCTCATTTTAATCCTCACTC	GTCTCCAACATGCCTCTCTTCA	CCGCTGGACCG CCATGTTTCTT	NM_017050
Gpx1	GGACATCAGGAGAATGGCAAGA	CGCACTTCTCAAACAATGTAAAGTTG	TTCCCTCAAGTATGTCCGACCCGGTG	NM_030826

## Data Availability

The data presented in this study are available on request.
